# Effect of exogenous glucoamylase on ruminal *in situ* and *in vitro* dry matter and starch degradability of cereal grains in beef cattle

**DOI:** 10.5713/ab.25.0328

**Published:** 2025-10-22

**Authors:** Lin Mu, Kleves V. Almeida, Carlos A. NinodeGuzman, Ignacio F. Marenchino, Kathy G. Arriola, Halima Sultana, Nicolas DiLorenzo, Wenting Li, Diwakar Vyas

**Affiliations:** 1 Department of Grassland Science, College of Agronomy, Hunan Agricultural University, Changsha, China; 2 College of Life Sciences and Agriculture, University of New Hampshire, Durham, NH, USA; 3 Department of Animal Sciences, Institute of Food and Agricultural Sciences, University of Florida, Gainesville, FL, USA; 4 Danisco Animal Nutrition and Health (IFF), Wilmington, DE, USA

**Keywords:** Beef, Glucoamylase, *In Vitro* Nutrient Degradability, Rumen Fermentation, Volatile Fatty Acids

## Abstract

**Objective:**

To evaluate the effects of glucoamylase on ruminal *in situ* and *in vitro* dry matter degradability (IVDMD), *in vitro* starch degradability (IVSD), volatile fatty acids (VFA), gas production and methane production using cereal grains as substrates in beef cattle.

**Methods:**

Six substrates (4 mm; 0.70 g per F57 bag) including sorghum (micronized and whole), barley (whole and steam-flaked), and corn (dry-rolled and steam-flaked); were incubated with exogenous glucoamylase (from *Trichoderma reesei*; 0.25 mg/g substrate dry matter [DM]) and buffered rumen fluid in six replicates per run in 3 independent runs. IVDMD, IVSD, *in situ* DMD (ISDMD), VFA, and methane production was measured after 7, and 12 h of incubation. ISDMD was measured after 0, 1, 3, 7, and 12 h of ruminal incubation in beef cattle fed high grain diets.

**Results:**

Exogenous glucoamylase increased IVDMD and gas production for all substrates except whole sorghum and barley while ISDMD was increased for all substrates except whole barley. Glucoamylase increased IVSD for steam flaked barley and corn. Glucoamylase supplementation increased molar proportion of propionate and decreased acetate-to-propionate ratio, regardless of substrates used. Similarly, glucoamylase supplementation increased methane production with steam-flaked corn.

**Conclusion:**

Glucoamylase supplementation can potentially improve DM and starch degradability in cereal grains commonly used as ingredients in beef cattle diets.

## INTRODUCTION

The livestock industry greatly relies on achieving the best possible nutritional value from feed ingredients including both forage and concentrates [1]. In beef cattle diets, starch serves as the primary energy source driving rapid weight gain during the finishing phase, primarily because it provides a readily fermentable carbohydrate that supports efficient muscle and fat deposition. Traditionally, starch is supplied through high-grain rations, which have been the cornerstone of feedlot finishing systems due to their ability to maximize growth rates and carcass quality in a relatively short period. Among cereal grains, barley, corn, and sorghum are primary ingredients for providing the main source of starch in livestock diets including beef cattle diets. However, the beef industry is increasingly facing scrutiny over such feeding practices. One concern centers on the ethical and economic implications of diverting large quantities of grain to livestock rather than direct human consumption. Another is the economic pressure from rising grain prices, which has heightened interest in strategies to optimize starch utilization. In light of these challenges, it is critical to maximize the efficiency with which starch is used in beef cattle diets, making any method that improves starch digestion and energy capture a valuable tool for both economic and environmental sustainability. Various processing methods including steam-flaking, micronizing, and dry-rolling are proven ways to improve the digestion and absorption of dietary starch and have gained prominence for increasing starch utilization and enhancing the overall efficiency of livestock production [2,3].

In addition to processing techniques, the use of exogenous amylase has displayed promise in enhancing the breakdown of starch in the rumen, increasing the degradability of diets, and ultimately enhancing lactational performance in dairy cows [4]. Several studies have reported increased organic matter (OM) degradability, and milk yield in response to amylase supplementation; however, results have been equivocal [4–7] and inclusion of exogenous amylase needs further research to optimize dietary conditions required for consistent efficacy. Moreover, the majority of the exogenous amylase application in ruminant studies used α-amylase [8] which primarily breaks down starch into oligosaccharides such as maltose and dextrins rather than glucose. In contrast, glucoamylases, enzymes from glycoside hydrolase family 15 (GH15) (EC 3.2.1.3), are exo-acting enzymes that hydrolyze starch from the non-reducing ends, releasing glucose as the final product. This distinction is critical because glucose is more readily fermentable in the rumen and may influence microbial protein synthesis. Despite their potential, the efficacy of glucoamylases on improving starch degradability and reducing methane (CH_4_) production from rumen fermentation is largely unexplored. Assessing the impact of enzyme supplementation on CH_4_ production is crucial, given that CH_4_ production can result in a loss of dietary energy, accounting for approximately 2% to 12% of the total gross energy intake in ruminant animals [9].

Recent studies have shown that the efficacy of glucoamylase may be influenced by grain processing methods, which alter starch structure and accessibility. Devant et al [10] reported improved starch digestibility in bulls fed rolled corn supplemented with glucoamylase, while Amaro et al [11] demonstrated that glucoamylase significantly enhanced *in vitro* starch digestibility in mature dent corn. These findings highlight the potential for substrate-specific responses to enzyme supplementation yet also underscore the knowledge gaps in understanding how processing level interacts with enzyme activity. The objective of this study was to evaluate the effect of exogenous glucoamylase supplementation on ruminal *in vitro* dry matter degradability (IVDMD), *in vitro* starch degradability (IVSD), and ruminal fermentation using different cereal grains as substrates incubated in rumen inoculum from beef cattle fed high grain diets. We hypothesized greater IVDMD, IVSD and *in situ* dry matter (DM) degradability of cereal grains with exogenous glucoamylase supplementation and lower CH_4_ production per unit of IVDMD, regardless of processing method used.

## MATERIALS AND METHODS

### Animal management and treatments

Three ruminal fluid donor steers were gradually transitioned to finishing diets (Table 1) and adapted to diets for a minimum of 14 d prior to the collection of ruminal fluid for *in vitro* batch culture and for conducting *in situ* studies. Substrates used for both *in vitro* and *in situ* study included 1) Micronized Sorghum, 2) Whole sorghum, 3) Whole barley, 4) Steam-flaked barley, 5) Dry-rolled corn, or 6) Steam-flaked corn with or without the inclusion of exogenous glucoamylase. Appropriate dilution of glucoamylase was prepared in 0.1 M citrate-phosphate buffer solution (pH 6.0) to achieve 0.25 mg of enzyme/g of substrate on a DM basis. The enzyme dilution was prepared no earlier than a day before incubation and was kept refrigerated at 4°C until application.

### *In vitro* batch culture

*In vitro* DM, starch, and neutral detergent fiber (NDF) degradability. *In vitro* batch culture study was conducted using serum vials for the estimation for IVDMD and IVSD while daisy incubator was used for the estimation of NDF degradability (IVNDFD). Rumen fluid was collected approximately 2 to 3 h after the morning feeding.

Rumen fluid was manually collected, filtered through two-layer cheesecloth, and pooled into pre-warmed thermos flasks kept at 39°C. Thermos flasks containing pooled rumen fluid were kept airtight until transported to the laboratory for final filtration with two more layers of cheesecloth (total of four layers). Rumen fluid inoculum was then added to a buffered pre-warmed (39°C) media Goering and Van Soest [12] media in a 1:2 ratio (rumen fluid: artificial saliva). The media was continuously infused with CO_2_ to maintain the anaerobic environment for the rumen fluid inoculum.

*In vitro* incubations were conducted on 3 separate days (runs) using the following substrates: 1) Micronized Sorghum, 2) Whole sorghum, 3) Whole barley, 4) Steam-flaked barley, 5) Dry-rolled corn, or 6) Steam-flaked corn with or without the inclusion of exogenous glucoamylase. Appropriate dilution of glucoamylase was prepared in 0.1 M citrate-phosphate buffer solution (pH 6.0) to achieve 0.25 mg of enzyme/g of substrate on a DM basis. The enzyme dilution was prepared no earlier than a day before incubation and was kept refrigerated at 4°C until application.

Substrates were ground to 4 mm and weighed into Ankom F57 filter bags (~0.70 g; Ankom Technology) and enzymes were applied directly to the substrate. Bags were sealed and immediately placed into serum bottles before incubation. No enzymes were applied to the control bags. Blank bags with no substrate were incubated to account for bag weight variation and gas production. Buffered rumen fluid (52 mL) was added to the 160-mL serum vials containing Ankom bags, and a continuous stream of CO_2_ was flushed into the vials during the whole inoculation process. Vials were closed with rubber stoppers and crimped with aluminum seals. Vials were immediately placed in an air-forced incubator at 39°C with a shaking system for 7 h, and 12 h. *In vitro* DM and starch degradability and gas production were examined using 6 replicates for each dose during a 7, and 12 h *in vitro* batch culture incubation in each of 3 runs. The sampling of 24-h micronized sorghum was compromised during experiment and data were not considered for analysis.

For the estimation of IVNDFD, 1,600 mL of buffered rumen inoculum was added to each of 4 Daisy jars. *In vitro* NDF degradability was observed for each substrate with or without addition of glucoamylase using 4 replicates for each enzyme dose in Daisy incubators for 12 and 24 h in each of 3 runs. Substrates used for IVNDFD were ground to 1 mm.

Daisy incubation was conducted using rumen fluid collected from ruminally cannulated beef cattle maintained on a high concentrate finishing diet. Rumen contents were obtained after morning feeding, strained through four layers of cheesecloth, and maintained under anaerobic conditions at 39°C. Daisy incubator was pre-set at 39°C. Ground feed samples (4 mm) were weighed into Ankom filter bags, sealed, and placed in Daisy II incubator jars containing buffer solution and rumen inoculum (ratio 1:4, v/v). The jars were flushed with CO_2_, sealed, and incubated at 39°C for 12 and 24 h incubation duration. Following incubation, the bags were rinsed thoroughly, dried at 60°C, and weighed to determine residue for *in vitro* NDF disappearance.

### *In situ* incubations

Three-ruminally fistulated steers (490±51 kg BW) transitioned to finishing diets were used for *in situ* incubations. For each set of incubations, substrates (micronized sorghum, whole sorghum, whole barley, steam-flaked barley, dry-rolled corn, or steam-flaked corn) were weighed into ANKOM F57 filter bags (~0.70 g; Ankom Technology). Several ANKOM bags were placed in large mesh retaining sacs which were attached to the cannula plug. These bags were incubated in the rumen for 0, 1, 3, 7 and 12 h. Samples were incubated in quadruplicate for each forage, treatment, and timepoint per steer resulting in 12 bags per combination. The bags were placed in the rumen via the cannula to measure the extent of DM digestion over time. All bags were simultaneously removed from the rumen and the cannula resealed. Regardless of bag size, no more than 300 g of samples were incubated at a time. Cattle had at least two days rest period before beginning another set of *in situ* incubation.

### Analytical procedures

#### Gas production

Gas pressure was measured with *in vitro* batch culture technique at 0, 2, 4, 7, and 12 h after incubation using a pressure transducer (model JYB-KO-MAG1; Omega Engineering) and subsequently converted to gas volumes after correcting for gas volumes from blank bottles. Gas production measurements were collected from serum bottles used for IVDMD and not from bottles used for IVSD. Based on the conditions in the laboratory, the following equation was used to convert pressure to volume:


Gas vol. (mL)=(Gas pressure [psi]×4.8843)+3.1296

Gas samples were collected from serum bottles after 7, and 12 h of incubation to determine methane production. Samples were collected in triplicates for each treatment and incubation duration combination.

*In vitro* DM degradability was calculated after 7, and 12 h of incubation (2 treatments×2 incubation durations×6 substrates×6 replicates) in 3 independent runs. The incubations were terminated by placing bottles on ice. Bags were taken out of serum bottles, washed with tap water until effluent was clear, dried in a forced-air oven set at 60°C for 48 h. Dried residues were weighed, and their DM concentrations were used to estimate IVDMD. *In vitro* starch degradability was estimated after 7, and 12 h incubation. Samples were incubated in 6 replicates for each incubation duration (2 enzymes× 2 incubation durations×6 substrates×6 replicates) in three independent runs. All bags were opened, and the residues were pooled to achieve enough sample for starch analysis. Substrates used for starch degradability were ground to 4 mm prior to incubations in the serum vials. Concentration of starch in samples was analyzed as described for Amaro et al [11].

#### pH, volatile fatty acids, and ammonia nitrogen analysis

The pH of the rumen inoculum post-incubation was measured using a pH meter (Corning Pinnacle M530; Corning). A 10-mL sample of the ruminal fluid was pooled into 15 mL centrifuge tubes and acidified with 0.1 mL of 20% H_2_SO_4_. A water-based solution using ethyl acetate extraction was used to determine VFA concentrations in the ruminal fluid samples. Samples were preparation, as described by Ruiz-Moreno et al [13] with a gas chromatograph (Agilent 7820A GC; Agilent Technologies) using a flame ionization detector and a capillary column (CP-WAX 58 FFAP 25 m×0.53 mm, Varian CP7767; Varian). Column temperature was maintained at 110°C, and the injector and detector temperatures were 200°C and 220°C, respectively [14]. Concentration of ammonia nitrogen (NH_3_-N) in samples was analyzed according to Broderick and Kang [15]. Samples were thawed at room temperature and centrifuged at 10,000×g for 15 min. The supernatant was analyzed using the phenol-hypochlorite method in a 96-well flat-bottom plate. Absorbance was measured with a spectrophotometer (SpectraMax Plus 384 Microplate Reader; Molecular Devices) at 620 nm.

#### Methane analysis

Methane concentration was analyzed using gas chromatography (Agilent 7820A GC; Agilent Technologies) with flame ionization detector and capillary column (Plot Fused Silica 25 m by 0.32 mm, Coating Molsieve 5A, Varian CP7536; Varian). Injector, column, and detector temperatures were maintained at 80°C, 160°C, and 200°C, respectively. Injector pressure was 20 psi with a total flow of 191.58 mL/min and a split flow of 185.52 mL/min with a 100:1 split ratio. Column pressure was 20 psi with a flow of 1.8552 mL/min. Detector makeup flow was 21.1 mL/min. The carrier gas was N_2_ and the run time was 3 min.

### Statistical analysis

Data were analyzed using the GLIMMIX procedure of SAS (ver. 9.1; SAS Institute). Each technique, *in vitro* batch culture, Daisy incubation, and *in situ*, was analyzed separately using appropriate factorial designs. *In vitro* batch culture technique was analyzed as a 6×2×2 factorial, with six substrate types, two treatment levels, and two incubation durations (7 h, 12 h). The model included substrate, treatment, and incubation duration as fixed effects, with run treated as a random blocking factor. Results are presented primarily for the substrate×treatment interaction, as the three-way interaction was not significant for most parameters. Where the three-way interaction was significant, detailed tables are provided in the supplementary section. For gas production data, treatment, sampling time, and their interaction were modeled as fixed effects. Sampling time was treated as a repeated measure, and the covariance structure was selected based on the lowest Akaike’s information criterion (AIC). Daisy incubation data were analyzed using a 6×2×2 factorial design, with 6 substrates, 2 treatments, and 2 incubation durations of 12 and 24 h. *In situ* technique followed a 6×2×5 factorial design, incorporating five incubation durations (0 h, 1 h, 3 h, 7 h, 12 h).

Prior to final analysis, residuals were evaluated for normality and outliers using the UNIVARIATE procedure in SAS. Statistical significance was declared at p<0.05, and trends were noted when 0.05≤p<0.10.

## RESULTS

The substrates used in this study are some of the most common ingredients used for beef cattle diets. The starch content for steam flaked corn (75.9%) and whole sorghum (74.7%) was greatest while whole barley (57.8%) and steam-flaked (54.6%) barley had numerically lowest starch content among all substrates. Micronizing sorghum and steam-flaking barley did not have any effect on the nutrient composition; however, steam-flaking corn lowered amylase-treated neutral detergent fiber (aNDF) and increased starch content (Table 2).

**Figure 1:**
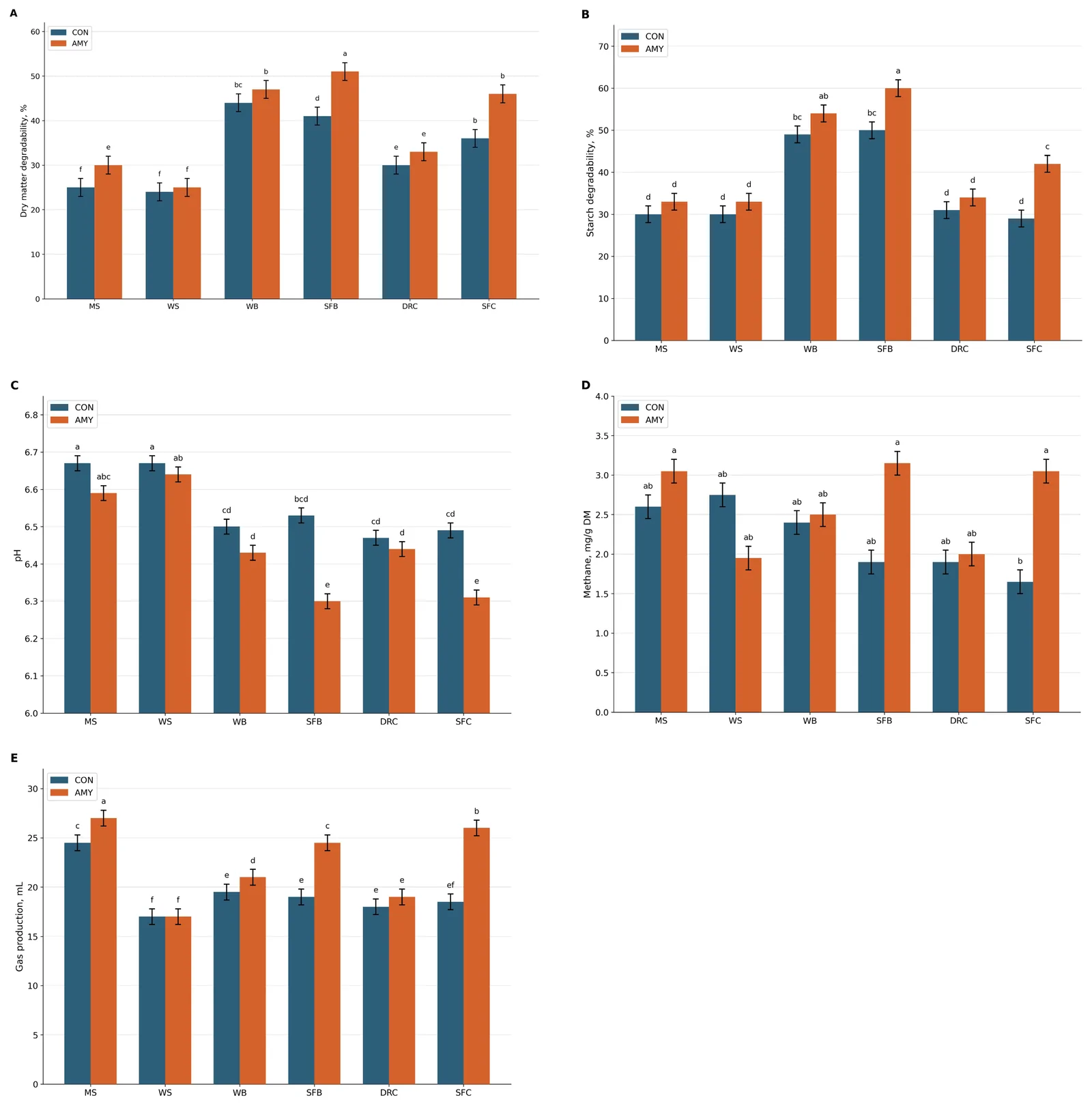
Effects of glucoamylase on (A) dry matter (DM) degradability (%), (B) starch degradability (%), (C) pH, (D) methane (mg/g DM), (E) gas production (mL) using different substrates incubated using rumen fluid from beef cattle fed high grain diets. Values were collected after 7, and 12 h on incubation duration. ^a–f^ Mean values on the bar chart with different superscripts differ according to the p-value for the treatment x substrate interaction. MS, micronized sorghum (only 7 h and 12 h); WS, whole sorghum; WB, whole barley; SFB, steam-flaked barley; DRC, dry-rolled corn; SFC, steam-flaked corn; CON, control; AMY, amylase.

**Figure 2:**
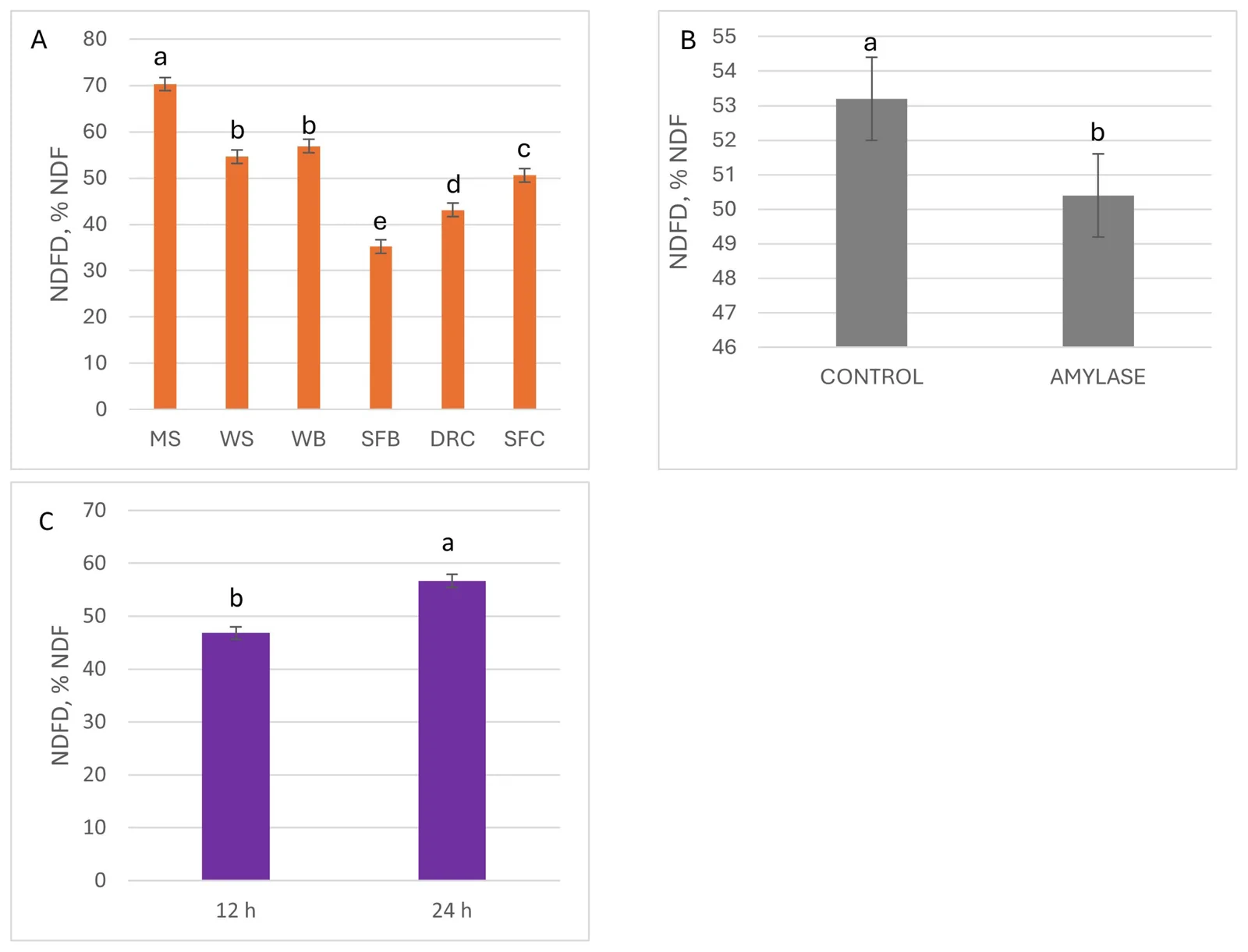
Effects of exogenous amylase on *in vitro* NDF degradability of various substrates measured using a Daisy incubator with rumen fluid collected from beef cattle fed high-grain diet. (A) Substrate. (B) Enzyme. (C) Hour. ^a–e^ Mean values on the bar chart with different superscripts differ according to the p-value for the substrate (A), treatment (B) or time (C) effect. MS, micronized sorghum; WS, whole sorghum; WB, whole barley; SFB, steam-flaked barley; DRC, dry rolled corn; SFC, steam-flaked corn; NDFD, neutral detergent fiber degradability; NDF, neutral detergent fiber.

Exogenous glucoamylase increased DM degradability during *in vitro* incubations for all substrates except whole sorghum and whole barley (p<0.01; Table 3; Figure 1A). The effects of exogenous glucoamylase on increasing IVSD were observed for steam-flaked corn and steam-flaked barley and the starch degradability was increased markedly (p<0.01; Table 3; Figure 1B) by 44.9% and 21.6%, respectively. No interaction effect was observed between substrate and glucoamylase on ammonia-N concentration; however, lower concentration was observed for steam-flaked barley, dry rolled corn, and steam-flaked corn compared to other substrates (Substrate effect; p = 0.03). Glucoamylase decreased rumen fluid pH and methane production with steam flaked barley and steam flaked corn compared to control; however, no changes were observed with other substrates (p<0.01; Figures 1C, 1D). Gas production was greater with glucoamylase for all substrates except whole sorghum (p<0.01; Figure 1E; Supplement 1). *In situ* DM degradability was greater for all substrates with glucoamylase supplementation except whole barley (Table 3; Figure 2A). In addition, regardless of type of substrates, glucoamylase increased DMD for every incubation duration (0 h, 3 h, 7 h, and 12 h) while no effects were observed when substrates were incubated for 1 h (p<0.01; Figure 2B).

**Figure 3:**
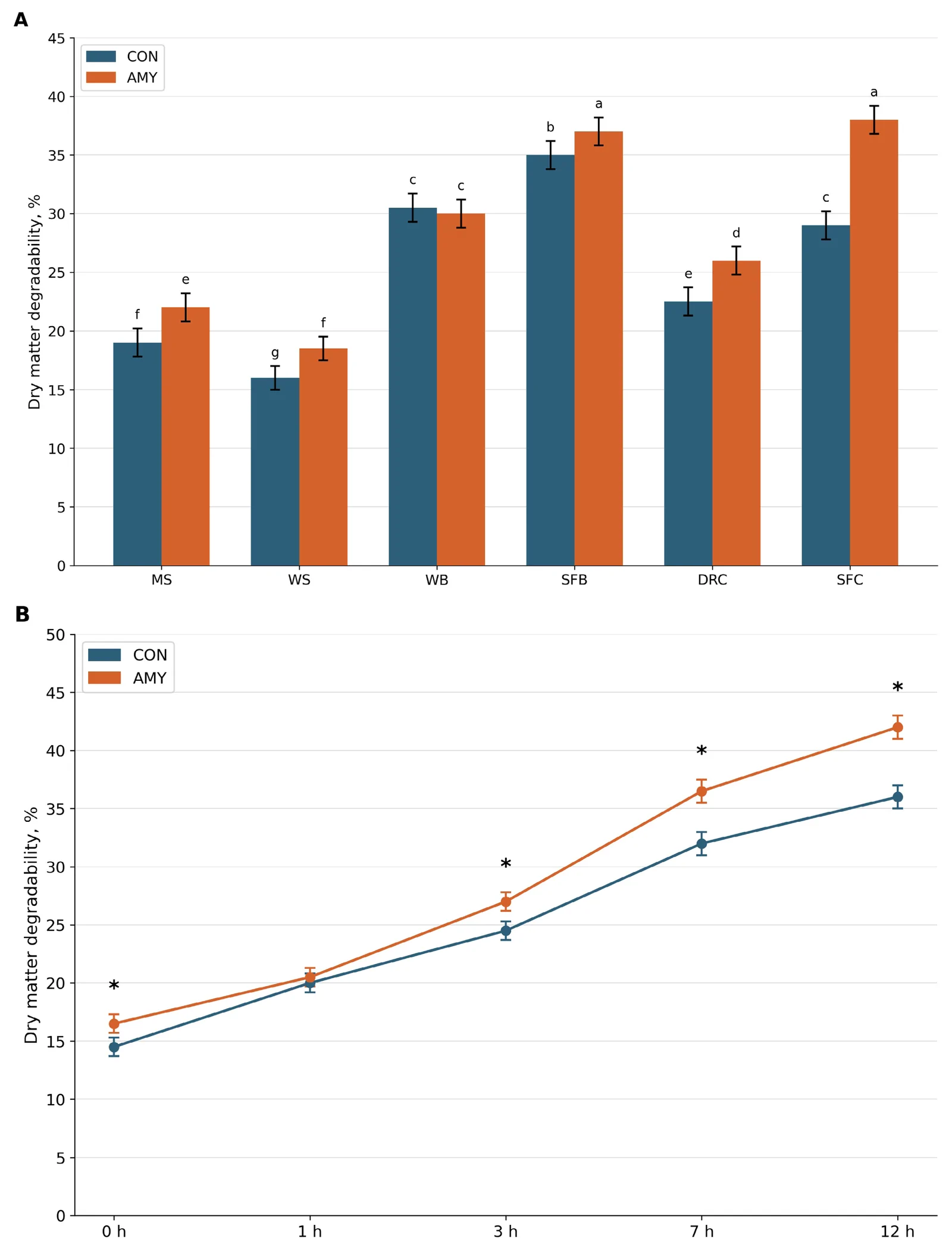
*In situ* DM degradability measured after ruminal incubation of different substrates incubation in cannulated beef cattle in response to (A) exogenous glucoamylase and (B) incubation duration of 0, 1, 3, 7, and 12 h. Glucoamylase: Exogenous glucoamylase from *Trichoderma reesei* was applied to each substrate at 0.25 mg/g DM. ^a–h^ Mean values on the bar chart with different superscripts differ according to the p-value for the treatment x substrate interaction (A) or time (B) effect. MS, micronized sorghum; WS, whole sorghum; WB, whole barley; SFB, steam-flaked barley; DRC, dry-rolled corn; SFC, steam-flaked corn; DM, dry matter; CON, control; AMY, amylase.

No interaction effects were observed on NDF degradability; however, micronized sorghum had greatest neutral detergent fiber degradability (NDFD) while steam flaked barley had lowest NDFD values (substrate effect: p<0.01; Figure 3A) while glucoamylase decreased NDFD (enzyme effect: p< 0.01; Figure 3B).

Glucoamylase supplementation tended to increase total VFA production regardless of the substrates used (p = 0.08); however, no interaction effects were observed (p = 0.71; Table 4). The molar proportion of acetate and acetate-to-propionate ratio was lower while molar proportion of propionate was greater (enzyme effect: p<0.01). While no enzyme effects were observed on butyrate (p = 0.89), valerate (p = 0.60), and isovalerate (p = 0.53). The molar proportion of iso-butyrate to be lower with amylase supplementation (p = 0.10). Total VFA production was similar for all substrates (p = 0.22); however, propionate production was lower with micronized sorghum compared with other substrates (p = 0.04). Butyrate (p<0.01) and valerate (p = 0.01) concentrations were lower for dry rolled corn compared with other substrates. Acetate-to-propionate ratio was greater with micronized sorghum compared with whole barley, dry rolled corn, and steam flaked corn (p = 0.05).

## DISCUSSION

Cereal grains have predominantly been used to supply starch for enhancing the energy density of ruminant diets. The extent to which starch from these grains can be digested and utilized plays a pivotal role in determining their nutritional value. Ruminal starch digestibility differs markedly among cereal grains [16–18]. The cereal endosperm cells are characterized by the presence of starch granules in the protein matrix, which is the primary factor affecting efficiency of starch utilization [19,20] with barley generally exhibiting the highest degradability, followed by corn and then sorghum. Similar results were observed in this study. Barley starch is highly fermentable in the rumen, with degradation rates often exceeding 90% under typical feeding conditions [21]. Corn starch shows moderate digestibility, which varies with processing; ground corn averages around 74%, while steam-flaked corn can reach up to 94% [21]. Sorghum starch is the least digestible in its native form, with ruminal degradation as low as 42%, primarily due to the dense protein matrix formed by kafirins that restrict microbial access [22]. Consequently, various mechanical and thermal processing techniques like micronization and steam-flaking have been employed to improve starch availability and digestion. Steam-flaking of corn has been shown to reduce aNDF and increase starch content, as observed in the present study, suggesting a separation of fibrous and starchy components during processing [23].

Exogenous enzyme preparations have a significant impact on feed degradation and also change the fermentation of the final product in the rumen, improving the efficiency of nutrient utilization [24]. The effects of exogenous glucoamylase were observed on ruminal DM degradability of every substrate except whole barley and whole sorghum while starch degradability was only increased for steam-flaked corn and barley. The effects on starch degradability with steam flaked grains may be attributed to increased surface area and reduced resistance to enzymatic hydrolysis. Schiff et al [25] demonstrated that supplementation with *Aspergillus oryzae* α-amylase (Amaize) increased the rate of gas production, a proxy for fermentability in steam-flaked corn, especially at higher flake densities. Similarly, Johnson et al [26] observed that steam-flaking barley at lower flake densities resulted in higher enzymatic starch reactivity and predicted ruminal starch digestion. These findings align with the observation that glucoamylase was effective in increasing starch degradability in steam-flaked corn and barley, but not in whole grains like barley and sorghum, which retain more intact structural barriers. The observed reduction in ruminal pH associated with steam-flaked grains further supports the enhanced fermentability of their starch fractions.

In addition to improvements in IVDMD, greater gas production and higher total VFA concentrations are typically expected, as these are direct end products of microbial fermentation [11]. In the present study, glucoamylase supplementation enhanced gas production across all substrates except whole sorghum, consistent with improvements in IVDMD. These findings align with previous reports where glucoamylase derived from *Trichoderma reesei*, *Aspergillus fumigatus*, and *Fusarium verticillioides* increased total gas volume when applied to mature dent corn compared to untreated controls [11]. Notably, CH_4_ production (expressed per gram of DM) also showed an increasing trend with glucoamylase supplementation [27], particularly in steam-flaked corn and steam-flaked barley. This is in agreement with the observed increases in IVDMD and IVSD, as CH_4_ is a byproduct of carbohydrate fermentation in the rumen. The enhanced CH_4_ output may also be linked to the enzymatic breakdown of starch into glucose, the primary end product of glucoamylase activity, rather than a mixture of glucose, maltose, and maltooligosaccharides typically produced by α-amylases. Supporting this, previous studies have shown that replacing refined starch with dextrose (glucose) increases intestinal CH_4_ production (g/d), suggesting that glucose availability may stimulate methanogenesis. The pronounced effects of glucoamylase in steam-flaked corn and barley likely reflect the greater enzymatic accessibility of starch in these processed grains, thereby enhancing fermentability and CH_4_ yield.

The changes observed in the molar proportions of VFA suggest that glucoamylase supplementation altered the fermentation dynamics of the substrates. Meale et al [27] reported a strong positive relationship between IVDMD and total VFA production, with species exhibiting higher IVDMD also producing greater VFA concentrations. In the present study, starch degradability increased, and ruminal pH decreased, both indicators of intensified microbial fermentation, total VFA concentration tended to increase with glucoamylase supplementation; however, no statistically significant changes were detected in total VFA concentrations between substrates and no interaction was observed between substrate and enzyme application. This may reflect limitations in analytical sensitivity or variability in microbial metabolic pathways under *in vitro* conditions. Notably, glucoamylase supplementation consistently increased the molar proportion of propionate and reduced the acetate-to-propionate ratio across all substrates and with glucoamylase supplementation. Propionate, a key substrate for gluconeogenesis, also contributes to ruminal pH regulation by consuming H+ ions. The shift toward propionate production suggests that glucose released from starch hydrolysis by glucoamylase was primarily fermented via the odd-chain VFA pathway [28]. This is consistent with findings from Nozière et al [29], who observed increased propionate molar proportions in first-lactation cows fed amylase derived from *Bacillus licheniformis* (McNeill et al [20]). Furthermore, Nozière et al [29] reported that high-starch diets supplemented with amylase altered VFA profiles, decreasing acetate while increasing propionate, isovalerate, valerate, and caproate. These shifts in VFA ratios, even in the absence of significant changes in total VFA concentration, reflect a meaningful change in fermentation pathways and microbial activity.

While we observed greater efficacy of glucoamylase in enhancing starch hydrolysis, the effects on lower NDFD were not expected. We can speculate that these effects may be attributed to shifts in microbial fermentation dynamics and ruminal conditions. Glucoamylase hydrolyzes starch into glucose, increasing the availability of rapidly fermentable carbohydrates leading to competitive suppression of fibrolytic bacteria such as *Ruminococcus albus* and *Fibrobacter succinogenes*, which are essential for fiber degradation. Additionally, greater fermentation of starch lowers ruminal pH, creating an environment less favorable for cellulolytic microbial activity, which typically declines when pH drops below 6.0; however, this may not be applicable in the present study since pH levels did not go below 6.0.

## CONCLUSION

Exogenous glucoamylase had significant impact on DM degradability and total gas production in most substrates, except for whole sorghum and barley. The enhancement in starch degradability, particularly with steam-flaked barley and corn, indicates the potential of glucoamylase to improve the nutritional value of cattle diets. However, the effect on CH_4_ production was variable, with an increase observed only in steam-flaked corn and barley. Amylase supplementation demonstrated a significant impact on total VFA production, and altered the molar proportion of VFAs suggesting that amylase can modify the fermentation profile, potentially leading to more efficient energy utilization in cattle diets. Overall, the study underscores the importance of substrate selection and processing methods in optimizing the nutritional content of beef cattle diets. The use of enzyme supplements, such as glucoamylase and amylase, can further enhance the degradability and fermentation characteristics of these diets, offering potential benefits for improving cattle nutrition.

## Figures and Tables

**Table 1:** Ingredient composition of step-up and finishing diets for cannulated steers

Ingredients (% DM)	Grazing	Step-1	Finishing diet
Bahiagrass pasture, grazing	100	-	-
Commodity blend pellets1)		50	
Cottonseed hulls	-	10	10
Corn grain, cracked	-	-	30
Corn gluten feed pellets	-	20	30
Citrus pulp pellets	-	20	25
Supplement2)	-	-	5

1) Commercial pellets made of cotton and peanut byproducts (AFG Feed).

2) Pelleted supplement custom-formulated to provide vitamins (A, D, E) and minerals, and to supply 35 mg of monensin and 10 mg of thiamine per kg of diet DM (Furst-McNess Company).

DM, dry matter.

**Table 2:** Nutrient composition of substrates used for *in vitro* batch culture and *in situ* incubations

Nutrient composition (%)	Micronized sorghum	Whole sorghum	Whole barley	Steam-flaked barley	Dry-rolled corn	Steam-flaked corn
DM (% fresh)	87.8	89.3	90.5	85.1	93.7	94.6
CP	11.6	11.2	12.4	11.1	9.3	8
aNDF	12.9	11.9	18.8	18.9	15.5	10.9
ADF	6.0	7.7	5.6	8.3	3.7	4.2
Starch	72.8	74.7	57.8	54.6	67	75.9

DM, dry matter; CP, crude protein; aNDF, amylase-treated neutral detergent fiber; ADF, acid detergent fiber.

**Table 3:** Effects of exogenous amylase on *in vitro* DM degradability, and rumen fermentation parameters at different incubation durations using different substrates

	Substrates (S)							Glucoamylase (E)1)			S	E	S×E
	
	MS	WS	WB	SFB	DRC	SFC	SEM	CON	AMY	SEM			
DMD (%)	27.5^d^	25.4^e^	45.9^a^	45.5^a^	31.3^c^	40.9^b^	1.60	33.7	38.8	1.50	<0.01	<0.01	<0.01
IVSD (%)	32.1^b^	32.3^b^	51.4^a^	54.4^a^	32.1^b^	35.7^b^	1.19	36.3	43.1	0.84	<0.01	<0.01	<0.01
Ammonia-N	8.11^a^	7.29^a^	8.03^a^	4.46^b^	5.17^b^	5.16^b^	1.08	7.02	5.71	0.73	0.03	0.11	0.80
pH	6.63^a^	6.67^a^	6.46^b^	6.41^b^	6.46^b^	6.39^b^	0.02	6.56	6.45	0.01	<0.01	<0.01	<0.01
Methane (mg/DM) incubated2)	2.82^a^	2.30^ab^	2.42^ab^	2.50^ab^	1.88^b^	2.31^ab^	0.35	2.18	2.56	0.31	0.04	0.02	<0.01
Gas production (mL)2)	26.5^a^	17.1^e^	20.2^c^	21.9^b^	18.2^d^	22.3^b^	0.78	19.3	22.8	0.77	<0.01	<0.01	<0.01
*In situ* DMD (%)3)	20.5	17.2	30.3	36.0	24.4	33.6	0.84	25.3	28.6	0.80	<0.01	<0.01	<0.01

1) Exogenous glucoamylase from *Trichoderma reesei* was applied to each substrate at 0.25 mg/g DM.

2) Methane and gas production values were collected from serum vials incubated for 7, and 12 h of incubation duration.

3) *In situ* DM degradability was measured at 0, 1, 2, 7, and 12 h in three cannulated beef cattle fed finishing diets.

a–e Mean values in the same row with different superscripts differ according to the p-value for the substrate or treatment effect.

DM, dry matter; MS, micronized sorghum; WS, whole sorghum; WB, whole barley; SFB, steam-flaked barley; DRC, dry-rolled corn; SFC, steam-flaked corn; SEM, standard error of the mean; DMD, dry matter degradability; IVSD, *in vitro* starch degradability.

**Table 4:** Effects of exogenous amylase on *in vitro* volatile fatty acids (VFA) concentration and molar proportions of individual VFA with different substrates

	Substrates (S)							Enzyme (E)			S	E	S×E
	
	MS	WS	WB	SFB	DRC	SFC	SEM	CON	AMY	SEM			
Total VFA (mM)	99.3	113.1	117.7	128.7	123.2	127.5	14.1	114.6	122.0	12.9	0.22	0.09	0.71
Individual VFA (mol/100 mol)													
Acetate	57.3	55.8	54.5	55.0	55.0	53.7	0.64	55.6^a^	54.8^b^	0.43	0.08	<0.01	0.97
Propionate	31.4^b^	33.0^ab^	34.0^ab^	34.0^ab^	34.7^a^	35.3^a^	1.06	33.9^a^	34.2^b^	0.84	0.04	<0.01	0.99
Butyrate	8.79^a^	8.35^ab^	8.50^a^	8.30^ab^	7.84^b^	8.32^ab^	0.34	8.36	8.34	0.30	<0.01	0.89	0.77
Isobutyrate	0.38	0.47	0.43	0.40	0.32	0.35	0.08	0.42	0.37	0.08	0.19	0.10	0.62
Valerate	1.18^b^	1.37^ab^	1.58^a^	1.32^ab^	1.27^b^	1.32^ab^	0.07	1.36	1.32	0.04	0.01	0.6	0.39
Isovalerate	0.99	1.07	1.03	0.94	0.89	0.99	0.12	1.03	0.94	0.11	0.12	0.53	0.77
A:P	1.83^a^	1.71^ab^	1.62^b^	1.64^ab^	1.60^b^	1.53^b^	0.06	1.70^a^	1.62^b^	0.05	0.05	<0.01	0.99

Volatile fatty acids were measured at different incubation durations (7 h, 12 h); however, no interaction was observed between substrate, enzyme, and incubation duration.

a,b Mean values in the same row with different superscripts differ according to the p-value for the substrate or treatment effect.

MS, micronized sorghum; WS, whole sorghum; WB, whole barley; SFB, steam-flaked barley; DRC, dry-rolled corn; SFC, steam-flaked corn; SEM, standard error of the mean.

## SUPPLEMENTARY MATERIAL

**Supplement 1**. Effects of glucoamylase on *in vitro* gas production at 0, 2, 4, 7, and 12 h incubation durations using different substrates.
