# Supplementary material for: Effect of exogenous glucoamylase on ruminal in situ and in vitro dry matter and starch degradability of cereal grains in beef cattle
Source: Anim Biosci. 2025 Oct 22;39(9):250328. doi: 10.5713/ab.25.0328 (PMC13547619; doi:10.5713/ab.25.0328)
Supplement: Supplementary file 1 [file ab-25-0328-Supplementary-1.pdf]

Supplement 1. Effects of glucoamylase on *in vitro* gas production at 0, 2, 4, 7, and 12 h incubation durations using different substrates

		Substrates[1)]													p-values[2)]		
		MS		WS		WB		SFB		DRC		SFC		SEM	S	A	S×A
		CON	AMY[3)]	CON	AMY	CON	AMY	CON	AMY	CON	AMY	CON	AMY				
Gas production (mL)	0	4.57	4.50	4.70	4.53	4.54	4.47	4.62	4.73	4.85	4.71	4.63	4.66	0.98	0.12	<0.01	0.15
	2	15.1[b]	16.7[a]	9.05[e]	8.73[e]	9.48[de]	10.7[cd]	8.88[e]	11.3[c]	8.20[e]	8.80[e]	8.61[e]	12.1[c]	0.98	<0.01	<0.01	<0.01
	4	23.3[b]	26.0[a]	13.4[ef]	13.6[ef]	14.8[de]	16.6[d]	15.1[de]	19.5[c]	12.4[f]	13.8[ef]	14.5[def]	21.0[c]	0.98	<0.01	<0.01	<0.01
	7	33.3[b]	37.9[a]	22.3[e]	22.8[e]	25.3[cde]	27.7[c]	26.3[cd]	33.3[b]	23.6[de]	25.2[cde]	23.9[de]	36.2[ab]	0.98	<0.01	<0.01	<0.01
	12	48.8[b]	55.0[a]	35.6[g]	36.8[fg]	42.4[cd]	46.0[bc]	40.6[def]	54.9[a]	38.3[efg]	42.1[cde]	38.8[defg]	58.6[a]	0.98	<0.01	<0.01	<0.01

[1)] Micronized Sorghum (MS), Whole Sorghum (WS), Whole Barley (WB), Steam-flaked Barley (SFB), Dry-rolled Corn (DRC), Steam-flaked Corn (SFC).

[2)] Substrate (S), Amylase (A), and Substrate×Amylase (S×A).

[3)] Exogenous glucoamylase from *Trichoderma reesei* was applied to each substrate at 0.25 mg/g DM.

[a–g] Mean values in the same row with different superscripts differ according to the p-value for the interaction effect for each time point.
